# Characteristics Associated with Early Worsening of Retinopathy in Patients with Type 2 Diabetes Diagnosed with Retinopathy at Their First Visit: A Retrospective Observational Study

**DOI:** 10.1155/2021/7572326

**Published:** 2021-07-17

**Authors:** Sayaka Wakabayashi Sugawa, Yoko Yoshida, Yusuke Hikima, Haruhiko Sato, Akira Shimada, Mitsuhiko Noda, Akifumi Kushiyama

**Affiliations:** ^1^Division of Diabetes and Metabolism, The Institute of Medical Science, Asahi Life Foundation, 2-2-6, Nihonbashi Bakurocho, Chuo-ku, Tokyo 103-0002, Japan; ^2^Department of Endocrinology and Diabetes, Saitama Medical University, 38 Morohongo Moroyama-machi, Iruma-gun, Saitama 350-0495, Japan; ^3^Department of Diabetes, Metabolism and Endocrinology, Ichikawa Hospital, International University of Health and Welfare, 6-1-14 Kounodai, Ichikawa City, Chiba 272-0827, Japan; ^4^Department of Pharmacotherapy, Meiji Pharmaceutical University, 2-522-1, Noshio, Kiyose City, Tokyo 204-8588, Japan

## Abstract

**Materials and Methods:**

Our study design was a retrospective observational study. Subjects with type 2 diabetes diagnosed with either simple or preproliferative diabetic retinopathy by ophthalmologists at their first visit and followed up for 6–18 months thereafter were included and divided into worsening and nonworsening groups. Thereafter, baseline characteristics and changes in HbA1c and therapy over a year were investigated.

**Results:**

Among the 88 subjects with simple diabetic retinopathy, 16% improved to no retinopathy, 65% retained their simple diabetic retinopathy, 18% worsened to preproliferative diabetic retinopathy, and 1% worsened to proliferative diabetic retinopathy. Among the 47 subjects with preproliferative diabetic retinopathy, 9% improved to simple diabetic retinopathy, 72% retained their preproliferative diabetic retinopathy, and 19% worsened to proliferative diabetic retinopathy. Patients with simple diabetic retinopathy had an odds ratio of 1.44 for worsening retinopathy with a 1% increase in baseline HbA1c. Meanwhile, the odds ratios for worsening retinopathy with a 1% decrease in HbA1c from baseline at 3, 6, and 12 months were 1.34, 1.31, and 1.38, respectively. Among patients with simple diabetic retinopathy, significantly more new interventions were introduced in the worsening group than in the nonworsening group.

**Conclusions:**

Increased baseline HbA1c, a substantial decrease in HbA1c, and intensified therapy were identified as risk factors for early worsening of diabetic retinopathy in patients with simple diabetic retinopathy at the first visit. Patients should therefore be intimately followed for retinopathy after their first visit.

## 1. Introduction

Diabetic retinopathy (DR) remains one of the major microvascular complications of diabetes, with severe cases possibly leading to blindness among adult patients. DR has two main stages, namely, nonproliferative (NPDR) and proliferative diabetic retinopathy (PDR) [1]. Accordingly, NPDR progresses from mild, moderate, and then severe [2], whereas the incidence of PDR increases as the baseline retinopathy stage worsens as shown in previous reports [3, 4]. The modified Davis classification [5–7] has commonly been used for grading retinopathy. Simple diabetic retinopathy (SDR) can be characterized by hard exudates, capillary aneurysms, or abnormal capillary aneurysm lesions. Meanwhile, preproliferative DR (PPDR) can be characterized by intraretinal hemorrhage, definite venous beading, definite intraretinal microvascular abnormalities, or soft exudates.

Studies have documented that the duration of diabetes [4], the high levels of HbA1c, blood pressure, and body mass index (BMI) [8], were risk factors for the onset and progression of DR. Compared with conventional glycemic control, intensive therapy has been known to reduce the progression of retinopathy in patients with both type 1 [9] and type 2 [10–13] diabetes. However, several reports have indicated that intensive therapy and/or substantial HbA1c reduction may be associated with early worsening of DR (EWDR) in patients with both type 1 [9, 14, 15] and type 2 [16–18] diabetes. Moreover, other specific and nonspecific risk factors for EWDR, unrelated to ordinary DR worsening, have been reported, including prolonged duration of diabetes, high baseline HbA1c levels, history of DR [3, 9, 14, 16, 17], and bariatric surgery [19, 20]. Furthermore, no current agreement exists regarding the appropriate timing of HbA1c reduction [21].

The current study retrospectively investigated whether the occurrence of EWDR in patients with type 2 diabetes diagnosed with SDR at their first visit differs according to baseline HbA1c, abruptness in HbA1c reduction, and treatment intensification.

## 2. Materials and Methods

### 2.1. Subjects

A total of 2334 patients with type 2 diabetes initially visited the Division of Diabetes and Metabolism, the Institute of Medical Science, Asahi Life Foundation (*n* = 2045), or the Department of Endocrinology and Diabetes, Saitama Medical University Hospital (*n* = 289) for glycemic control or diabetic education from January 2006 through October 2015. The subjects were evaluated for retinopathy either at the Institute of Medical Science, Asahi Life Foundation, or at the Saitama Medical University Hospital. Ophthalmologists observed and sketched the entire area with mydriasis, and retinopathy was classified into four stages of severity according to the modified Davis classification: no retinopathy, SDR, PPDR, proliferative retinopathy (PDR), or photocoagulation. Fundus photographs were obtained when there was a change in fundus findings or when fluorescein angiography was performed. Subjects were classified as SDR by the presence of hard exudates, capillary aneurysm, or abnormal capillary aneurysm lesions, and they were classified as PPDR by the presence of intraretinal hemorrhage, definite venous beading, definite intraretinal microvascular abnormalities, or soft exudates. We included subjects diagnosed with SDR or PPDR following ophthalmologic examinations within 6 months after their first visit and those who underwent fundus examinations two or more times within 6–18 months after their first examination. Among these subjects, those who did not undergo the first fundus examinations prior to HbA1c measurement 3 months after the first visit or the last fundus examination after HbA1c measurement at 12 months were excluded. When differences in the classification of both eyes were present at the first visit of patients, the worse one was used. Worsening retinopathy was defined as disease stage progression after the first visit. For those who visited ophthalmologic clinics several times after their first visit, findings during the period closest to 12 months after their first visit were used.

### 2.2. Laboratory Tests and Statistical Analysis

Baseline demographic information, such as sex, age, disease duration, blood pressure, BMI, biochemical profiles, and use of statins and antihypertensive agents, was collected as previously described [22]. Previous medications taken before the first visit and new interventions provided were also determined.

Patients were categorized into four groups according to previous medications taken before their first visit: “none,” “nonsulfonylurea (non-SU),” sulfonylurea (SU),” and “insulin,”, respectively. “None” indicates patients taking neither oral hypoglycemic agents nor insulin. “Non-SU” indicates those taking antidiabetics except for insulin, SU, or glinides. “SU” indicates those taking SU or glinide, including combinations of other hypoglycemic agents, except for insulin. “Insulin” indicates those taking insulin, including any combination of hypoglycemic agents. Patients were also categorized according to new interventions received at their first visit based on the treatment stages of their previous medications. For instance, when “non-SU” patients received other non-SU agents as their new intervention, the new intervention was categorized as “none” given that no treatment intensification on the subsequent stage occurred. Moreover, when patients with no prior medication received insulin and glinide, the new intervention was categorized as “insulin.” Therapy intensification was defined as “yes” when any new intervention was confirmed and “no” when no new interventions were received.

HbA1c levels were assessed at the first visit, as well as after 3, 6, and 12 months: baseline HbA1c, 3 M HbA1c, 6 M HbA1c, and 12 M HbA1c, respectively. Thereafter, the magnitude of HbA1c reduction 3, 6, and 12 months after the first visit was determined by subtracting baseline HbA1c levels from 3 M HbA1c, 6 M HbA1c, and 12 M HbA1c, with HbA1c reduction being indicated by “*Δ*” (*Δ*3 M HbA1c, *Δ*6 M HbA1c, and *Δ*12 M HbA1c, respectively). To assess HbA1c reduction during the follow-up period, the magnitude of HbA1c reduction at 3 and 6 months (*Δ*3 M HbA1c and *Δ*6 M HbA1c) was subtracted from that at 12 months (*Δ*12 M HbA1c): *Δ*12 M-*Δ*3 M HbA1c and *Δ*12 M-*Δ*6 M HbA1c.

After the aforementioned procedures, multiple logistic regression analysis adjusting for age, sex, BMI, and use of antihypertensive agents was performed by using retinopathy progression as the objective variable and baseline HbA1c, *Δ*3 M HbA1c, *Δ*6 M HbA1c, *Δ*12 M HbA1c, and treatment profiles as explanatory variables in patients with SDR to calculate the odds ratios.

Retinopathy progression was evaluated by dividing patients with SDR into two groups according to retinopathy findings after treatment initiation, namely, worsening and nonworsening groups. Both groups were then compared in terms of other collected variables.

This study was approved by the Ethics Committees of both the Institute of Medical Science, Asahi Life Foundation (approval number 10302-5-A), and Saitama Medical University Hospital (approval number 18123.03). All clinical investigations were conducted in accordance with the tenets of the Declaration of Helsinki. All statistical analyses were performed using JMP version 14.2 (SAS Institute Inc.), with *p* < 0.05 indicating statistical significance during regression analysis.

## 3. Results

According to the results of the fundus examination, we obtained 109 SDR and 63 PPDR subjects, and 88 SDR and 47 PPDR subjects could be included in the analyses of the comparison between EWDR and patients' characteristics, including HbA1c changes, as per the inclusion criteria.  Table 1 summarizes the patients' characteristics at their first visit. Accordingly, patients with SDR and PPDR, 80% and 79% of whom were male, had a mean age of 58 ± 10 and 52 ± 8 years, diabetes duration of 9 (4, 15) and 9 (3, 15) years, BMI (kg/m^2^) of 25.0 ± 3.8 and 25.8 ± 4.9, percentage of newly diagnosed diabetes of 14% and 6%, baseline HbA1c of 9.1% ± 2.0% (range 5.9%–13.9%) and 9.5% ± 1.9% (range 5.6%–13.9%), *Δ*3 M HbA1c of −1.7% ± 1.8% (range −7.5%–1.2%) and −1.9% ± 1.9% (range −7.3%–1.2%), *Δ*6 M HbA1c of −1.9% ± 2.0% (range −8.8%–1.5%) and −2.2% ± 1.9% (range −6.4%–1.1%), *Δ*12 M HbA1c of −1.9% ± 2.0% (range −8.4%–0.9%) and −2.1% ± 1.8% (range −6.3%–0.9%), and baseline urinary albumin creatinine ratio of 12 (7, 36) and 29 (11, 110) mg/gCre, respectively. Among subjects with SDR at their first visit, 16% (14 patients) improved to no retinopathy, 65% (57 patients) retained their SDR, 18% (16 patients) worsened to PPDR, and 1% (one patient) worsened to PDR. Meanwhile, among those with PPDR at their first visit, 9% (4 patients) improved to SDR, 72% (34 patients) retained their PPDR, and 19% (9 patients) worsened to PDR.

Logistic regression analysis for the worsening (17 patients) and nonworsening groups (71 patients) revealed an odds ratio of 1.44 for the worsening retinopathy with a 1% increase in baseline HbA1c level (Table 2). Moreover, the odds ratios for worsening retinopathy with a 1% decrease in *Δ*3 M HbA1c, *Δ*6 M HbA1c, and *Δ*12 M HbA1c were 1.34, 1.31, and 1.38, respectively. The odds ratios for worsening retinopathy with a 1% decrease in *Δ*12 M-*Δ*3 M HbA1c and *Δ*12 M-*Δ*6 M HbA1c were 2.04 and 2.96, respectively. The cutoff values of *Δ*3 M HbA1c, *Δ*6 M HbA1c, *Δ*12 M HbA1c, *Δ*12 M-*Δ*3 M HbA1c, and *Δ*12 M-*Δ*6 M HbA1c maximized the sum of sensitivity plus specificity −1 (Youden index) for worsening retinopathy were −1.6% (area under the curve 0.71), −1.5% (0.69), −1.6% (0.75), −0.6% (0.67), and −0.4% (0.73), respectively. These cutoff values remained almost constant regardless of the duration of HbA1c decrease. Moreover, those not taking antihypertensive agents exhibited significantly greater retinopathy worsening. No associations were observed between retinopathy progression and sex, statin use, baseline blood pressure, lipid, creatinine, uric acid levels, and urinary albumin creatinine ratio.

**Figure 1:**
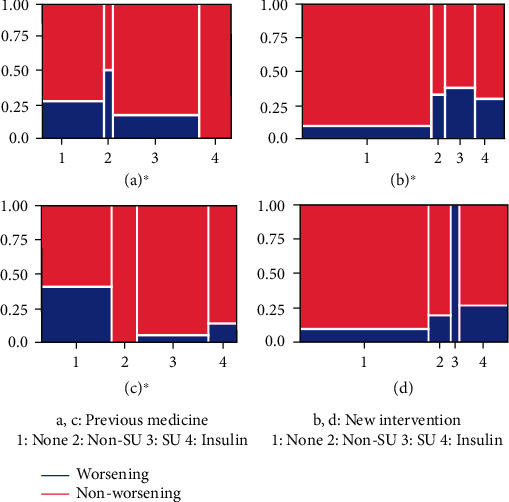
Rates of previous medication and new interventions in a patient with simple diabetic retinopathy (a, b) and preproliferative diabetic retinopathy (c, d). Cochran–Armitage trend test was performed, with *p* < 0.05 indicating statistical significance (∗). The *X*-axis classifies patients into four categories: none (1), nonsulfonylurea (2), sulfonylurea (3), or insulin (4). The width represents the number of patients classified. The *Y*-axis indicates the number of patients classified into two categories: worsening (blue) or nonworsening (red). Among patients with SDR, those who received no previous medication tended to have worsening retinopathy (a). New intervention tended to significantly intensify in worsening patients with SDR (b). Among patients with PPDR, those who received no previous medication tended to have worsening retinopathy (c).

The relationship between EWDR and previous medication or new intervention determined using the Cochran–Armitage trend test is presented in Figure 1. Among patients with SDR or PPDR, those who did not receive previous medications tended to have worsening retinopathy. Among patients with SDR, the new intervention tended to significantly intensify in the worsening group, whereas among patients with PPDR, the new interventions did not differ between both groups. Multiple logistic regression analysis was then utilized by using retinopathy progression as the objective variable and baseline HbA1c, *Δ*3 M HbA1c, *Δ*6 M HbA1c, *Δ*12 M HbA1c, and therapy intensification as explanatory variables among patients with SDR (Table 3). Accordingly, *Δ*12 M HbA1c and baseline HbA1c were determined to be significantly associated with EWDR after adjusting for age, sex, BMI, and use of any antihypertensive agents. *Δ*3 M HbA1c, *Δ*6 M HbA1c, and *Δ*12 M HbA1c were not independent of therapy intensification and/or baseline HbA1c for EWDR, and odds ratios of *Δ*3 M HbA1c and *Δ*6 M HbA1c were changed from more than 1.00 to less than 1.00. Moreover, the decrease in HbA1c, baseline HbA1c, and therapy intensification were confounded with each other for EWDR.

**Figure 2:**
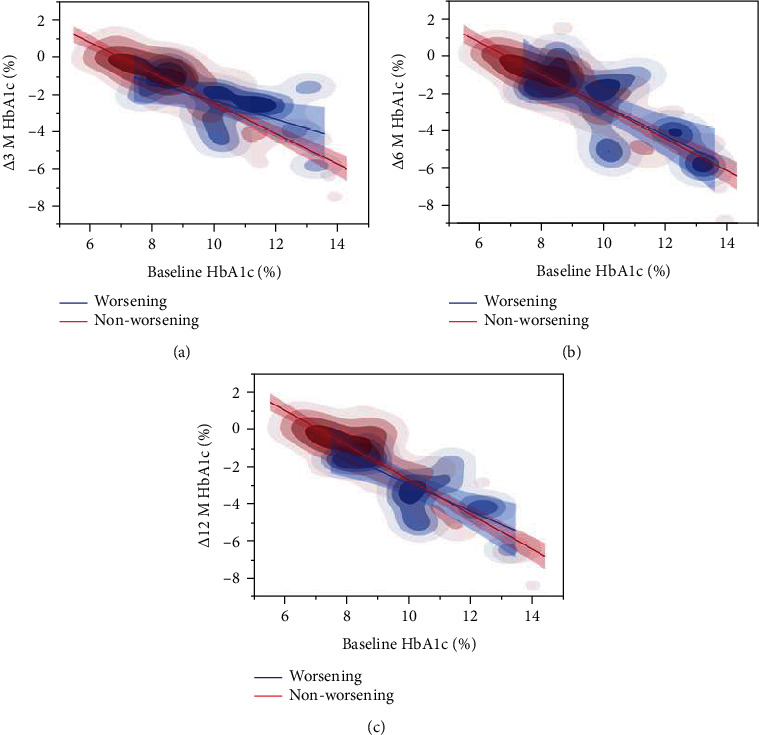
Two-dimensional density plots and regression line of baseline HbA1c and *Δ*HbA1c comparing the retinopathy worsening (blue) and nonworsening (red) groups in patients with simple diabetic retinopathy (SDR). The *X*-axis represents baseline HbA1c level, whereas the *Y*-axis represents *Δ*3 M HbA1c (a), *Δ*6 M HbA1c (b), or *Δ*12 M HbA1c (c). High baseline HbA1c was associated with lower levels of *Δ*3 M HbA1c, *Δ*6 M HbA1c, and *Δ*12 M HbA1c. The worsening group had greater baseline HbA1c distribution at the high region and greater *Δ*3 M HbA1c, *Δ*6 M HbA1c, and *Δ*12 MHbA1c distribution at the low region compared to the nonworsening group.

Figure 2 shows the correlation between baseline HbA1c and *Δ*HbA1c in worsening and nonworsening patients with SDR. Accordingly, high baseline HbA1c levels were found to be associated with lower *Δ*3 M HbA1c, *Δ*6 M HbA1c, and *Δ*12 M HbA1c similarly between worsening and nonworsening subjects. However, the distribution density showed that the worsening group had greater baseline HbA1c distribution at the high region and greater *Δ*3 M HbA1c, *Δ*6 M HbA1c, and *Δ*12 M HbA1c distribution at the low region compared to the nonworsening group.

## 4. Discussion

The current study showed that a certain proportion of patients with type 2 diabetes who were diagnosed with SDR or PPDR at the first visit developed EWDR within one year. Moreover, our results showed that reduced HbA1c, intensification of hypoglycemic pharmacotherapy, and baseline HbA1c levels were associated with EWDR in subjects with SDR. Unfortunately, no previous studies have determined the rate of progression from SDR to PPDR, while the incidence of PDR in patients with PPDR observed herein was similar to that presented in previous reports [23, 24].

No current agreement exists on the timing of HbA1c reduction to prevent EWDR [21]. Nonetheless, the present study found that the relationship between EWDR and HbA1c reduction was independent of the timing of HbA1c reduction, considering that the cutoff value of HbA1c reduction over time for retinopathy worsening remained almost constant. Accordingly, our findings suggest that a 1.6% decrease in HbA1c at any time within 12 months since the first visit could be a risk factor for EWDR. No difference in the ratio of baseline HbA1c to HbA1c reduction during hospital visits had been noted between patients with EWDR and those who showed no retinopathy progression. Given the correlation between baseline HbA1c levels and HbA1c decrease, determining which variable contributed more to EWDR remains challenging.

Among patients with SDR, EWDR increased when no previous medication or new pharmacotherapy interventions were introduced. However, the new intervention for EDWR was not independent of HbA1c and *Δ*HbA1c. Treatment selection may have been confounded with HbA1c reduction. It should be noted that 27% of the subjects who did not receive previous medications for SDR were newly diagnosed with diabetes; although it was unanalyzable, this high percentage of the subjects presumably influenced the detected significance of the previous medication. Meanwhile, among patients with PPDR, previous medication, and not therapy intensification, affected EWDR, indicating that the retinopathy onset might have preceded the first diagnosis. This speculation is supported by the considerably short diabetes duration among patients with PPDR having EWDR, who might have experienced rapid progression of retinopathy and/or a long interval of untreated diabetes. Although several studies have reported retinopathy worsening following insulin injection [16, 17, 25], the mechanism underlying such a development has remained unclear [26]. However, previous research has hypothesized [26] that exogenous insulin acts synergistically with vascular endothelial growth factor expressed by the ischemic retina, thereby triggering vascular proliferation and worsening of diabetes retinopathy. Nonetheless, the mechanisms behind EWDR have yet to be elucidated [21]. Studies have shown that numerous cytokines, namely, growth hormones, insulin-like growth factor-1 [27, 28], vascular endothelial growth factor [29], and erythropoietin [30], are involved in DR.

Hypoglycemia often occurs when intensive glycemic control is achieved [12] with reports suggesting a relationship between hypoglycemia and worsening of retinopathy [31, 32]. However, other studies have also reported that hypoglycemia was not associated with retinopathy [33] and that intensive treatment reduced microvascular complications despite increased hypoglycemia [12]. Granting that a relationship exists between EWDR and hypoglycemia, new interventions, such as SU and insulin, could cause EDWR through hypoglycemia owing to their pharmacological mechanisms. However, subjects categorized as non-SU, who received neither SU nor glinide nor insulin, tended to suffer from EDWR. Therefore, hypoglycemia may not be the only cause for EDWR. A double-blind trial [34, 35] revealed that the GLP-1 analog semaglutide promoted more DR complications compared to placebo among high-risk patients. Given that the GLP-1 analog apparently does not increase hypoglycemia, DR worsening was suggested to have been caused by preexisting DR and the rapid improvement in glycemic control.

Studies [4] and guidelines [36] recommend a retinopathy follow-up interval of 6 months to 2 years among patients who have mild NPDR given the increased incidence of retinopathy requiring treatment when the fundus examination interval exceeds 2 years [37]. Frequent follow-up is required in high-risk groups with more advanced DR and high HbA1c levels [38]. Therefore, patients with high HbA1c levels and SDR at their first visit should be referred to an ophthalmologist within 1 year.

Previous randomized controlled trials (RCTs) [9, 14, 15] involving patients with type 1 diabetes have identified high baseline HbA1c, rapid improvement in glycemic control, history of DR, intensified treatment, long diabetic duration, women, and pregnancy as risk factors for EWDR [39, 40]. Unfortunately, no large RCTs have been conducted on patients with type 2 diabetes [21], although previous non-RCTs [16–18, 41] have shown that high baseline HbA1c, rapid improvement in glycemic control, intensified treatment, previous DR, long diabetic duration, and bariatric surgery were risk factors for EWDR among those with type 2 diabetes [19, 20]. Moreover, a previous report [15] found that baseline HbA1c exceeding 10.1% increased the risk of EWDR in both intensive and conventional treatment groups. Therefore, patients with high HbA1c at their first visit need to be mindful of EWDR regardless of treatment. Although duration of diabetes has been identified as a risk factor for EWDR [15, 41], the current study found no significant relationship between duration of diabetes and EWDR, with 14% (12 patients) of patients with SDR having been newly diagnosed with type 2 diabetes. Therefore, the duration of diabetes could have been underestimated. Similar to the previous studies [21], the current study found that blood pressure or lipids were not associated with EWDR.

Some limitations of the current study are worth noting. Firstly, the number of subjects was relatively small. Due to the limited number of subjects with PPDR, a detailed analysis could not be performed. Moreover, no difference in the characteristics of EWDR was observed between those with SDR and PPDR. A previous study [15] showed that EWDR occurred in 13% and 7.6% of those with type 1 diabetes who received intensive and conventional treatment, respectively. Based on such conditions, at least 962 subjects were required to satisfy a statistical power of 80%. Therefore, the several subanalyses conducted herein might have diminished significance due to the small number of subjects. Secondly, various biases inherent to retrospective studies may have been present. For instance, treatment bias may have occurred among physicians who were already knowledgeable regarding the relationship between rapid improvement in glycemic control and EWDR. However, our dataset comprising clinical practice information from several certified diabetologists across Japan showed that HbA1c reduction was not hindered in accordance with EWDR information. Finally, the short follow-up period is a limitation of this study. If the follow-up period had been 2 years or more, the progress of retinopathy may have been found in more patients.

## 5. Conclusion

Although initiating or changing therapy can effectively improve glycemic control, rapid glycemic control has been associated with EWDR. The current study identified high baseline HbA1c and a large decrease in HbA1c as risk factors for EWDR among patients diagnosed with SDR at their first visit. As such, patients should be closely followed up for retinopathy within a year after their first visit, regardless of the decline of HbA1c levels and the type of hypoglycemic agents administered.

## Figures and Tables

**Table 1:** Summary of the clinical characteristics of patients with simple and preproliferative diabetic retinopathy.

	SDR			PPDR		
	Total	Nonworsening	Worsening	Total	Nonworsening	Worsening
	(*n* = 88)	(*n* = 71)	(*n* = 17)	(*n* = 47)	(*n* = 38)	(*n* = 9)
Age (years)	58 ± 10	59 ± 10	54 ± 11	52 ± 8	52 ± 8	53 ± 9
Sex (% male)	80	76	94	79	82	67
Duration of diabetes (years)	9 < 4, 15>	10 < 4, 17>	7 < 1, 11>	9 < 3, 15>	9 < 4, 15>	7 < 1, 13>
Body mass index (kg/m^2^)	25.0 ± 3.8	25.2 ± 3.8	24.1 ± 3.6	25.8 ± 4.9	26.1 ± 5.3	24.6 ± 2.4
Newly diagnosed diabetes (%)	14	13	18	6	5	11
Previous medication (%) None/non-SU^†^/SU^‡^/insulin	33/5/45/17	30/3/46/21	47/12/41/0	36/13/36/15	26/16/42/16	78/0/11/11
New intervention (%) None/non-SU^†^/SU^‡^/insulin	64/6/15/15	70/6/11/13	35/12/29/24	62/11/4/23	68/11/0/21	33/11/22/33
Therapy intensification (% yes)	36	30	65	38	32	67
Use of statins (%)	28	30	24	36	37	33
Use of antihypertensive agents (%)	53	59	29	51	45	78
Baseline HbA1c (%)	9.1 ± 2.0	8.9 ± 1.9	10.3 ± 1.8	9.5 ± 1.9	9.4 ± 1.9	9.8 ± 2.3
3 M HbA1c (%)	7.4 ± 1.1	7.3 ± 0.9	7.8 ± 1.5	7.6 ± 1.2	7.8 ± 1.3	7.0 ± 0.7
6 M HbA1c (%)	7.2 ± 1.1	7.1 ± 1.0	7.4 ± 1.4	7.3 ± 1.1	7.4 ± 1.0	6.9 ± 1.2
12 M HbA1c (%)	7.1 ± 1.0	7.1 ± 0.9	7.0 ± 1.2	7.4 ± 1.1	7.5 ± 1.1	6.8 ± 0.9
*Δ*3 M HbA1c (%)	−1.7 ± 1.8	−1.5 ± 1.8	−2.5 ± 1.5	−1.9 ± 1.9	−1.7 ± 1.8	−2.8 ± 2.2
*Δ*6 M HbA1c (%)	−1.9 ± 2.0	−1.7 ± 1.9	−2.9 ± 2.0	−2.2 ± 1.9	−2.1 ± 1.7	−2.9 ± 2.6
*Δ*12 M HbA1c (%)	−1.9 ± 2.0	−1.7 ± 2.0	−3.1 ± 1.7	−2.1 ± 1.8	−1.9 ± 1.6	−3.0 ± 2.2
Baseline systolic blood pressure (mmHg)	137 ± 18	138 ± 19	135 ± 16	142 ± 24	142 ± 23	142 ± 29
Baseline diastolic blood pressure (mmHg)	81 ± 14	81 ± 14	83 ± 11	85 ± 15	86 ± 15	81 ± 15
Baseline triglyceride (mg/dL)	146 < 104, 239>	164 < 104, 243>	137 < 89, 163>	124 < 85, 173>	131 < 85, 198>	102 < 78, 148>
Baseline total cholesterol (mg/dL)	210 ± 63	212 ± 64	203 ± 56	208 ± 45	206 ± 49	217 ± 24
Baseline high density lipoprotein (mg/dL)	51 ± 13	52 ± 14	50 ± 12	55 ± 14	54 ± 14	60 ± 11
Baseline low density lipoprotein (mg/dL)	116 ± 36	113 ± 30	126 ± 55	122 ± 41	123 ± 44	120 ± 19
Baseline non-high-density lipoprotein (mg/dL)	159 ± 63	161 ± 65	152 ± 57	153 ± 44	152 ± 47	156 ± 26
Creatinine (mg/dL)	0.8 ± 0.3	0.8 ± 0.3	0.7 ± 0.2	0.7 ± 0.2	0.7 ± 0.2	0.7 ± 0.2
Uric acid (mg/dL)	5.2 ± 1.2	5.2 ± 1.1	5.1 ± 1.4	5.4 ± 1.3	5.4 ± 1.4	5.5 ± 0.9
Urinary albumin/creatinine ratio (mg/gCre)	12 < 7, 36>	11 < 6, 32>	13 < 11, 50>	29 < 11, 110>	29 < 11, 122>	35 < 16, 99>

Variables are expressed as mean ± standard deviation or median < 1^st^ quartile, 3^rd^ quartile>. ^†^Nonsulfonylurea. ^‡^Sulfonylurea.

**Table 2:** Univariate logistic regression analysis for retinopathy progression in patients with simple diabetic retinopathy.

	OR	95% CI	*p* value	AUC	Cutoff value
Age (+1 years)	0.95	0.90–1.00	0.09		
Sex (males)	5.03	0.92–94	0.13		
Diabetes duration (+1 years)	0.93	0.85–0.99	0.05		
Body mass index (+1 kg/m^2^)	0.92	0.79–1.06	0.28		
Newly diagnosed diabetes (yes)	1.48	0.30–5.72	0.59		
New intervention (nonsulfonylurea vs. none)	4.17	0.50–26.8	0.16		
New intervention (sulfonylurea vs. none)	5.21	1.25–21.7	0.02^*^		
New intervention (insulin vs. none)	3.70	0.81–15.8	0.09		
Therapy intensification (yes)	4.37	1.47–14.2	0.01^*^		
Use of statins (yes)	0.73	0.19–2.35	0.62		
Use of antihypertensive agents (yes)	0.29	0.08–0.86	0.03^*^		
Baseline HbA1c (+1%)	1.44	1.10–1.94	0.01^*^	0.73	9.8
3 M HbA1c (+1%)	1.51	0.92–2.54	0.10		
6 M HbA1c (+1%)	1.25	0.75–2.07	0.39		
12 M HbA1c (+1%)	0.90	0.49–1.60	0.74		
*Δ*3 M HbA1c (−1%)	1.34	1.00–1.82	0.05	0.71	−1.6
*Δ*6 M HbA1c (−1%)	1.31	1.00–1.74	0.048^*^	0.69	−1.5
*Δ*12 M HbA1c (−1%)	1.38	1.06–1.83	0.02^*^	0.75	−1.6
*Δ*12 M-*Δ*3 M HbA1c (−1%)	2.04	1.12–4.30	0.03^*^	0.67	−0.6
*Δ*12 M-*Δ*6 M HbA1c (−1%)	2.96	1.34–6.96	0.01^*^	0.73	−0.4
Baseline systolic blood pressure (mmHg)	0.99	0.96–1.02	0.61		
Baseline diastolic blood pressure (mmHg)	1.01	0.97–1.05	0.60		
Baseline triglyceride (mg/dL)	1.00	0.99–1.00	0.16		
Baseline total cholesterol (mg/dL)	1.00	0.99–1.01	0.57		
Baseline high density lipoprotein (mg/dL)	0.99	0.95–1.03	0.70		
Baseline low density lipoprotein (mg/dL)	1.01	0.99–1.03	0.25		
Baseline non-high-density lipoprotein (mg/dL)	1.00	0.98–1.01	0.61		
Creatinine (mg/dL)	0.27	0.02–2.28	0.30		
Uric acid (mg/dL)	0.91	0.57–1.42	0.67		
Urinary albumin/creatinine ratio (mg/gCre)	1.00	0.99–1.00	0.91		

^*^ *p* < 0.05.

**Table 3:** Multivariate logistic regression analysis of possible factors for retinopathy progression in patients with simple diabetic retinopathy.

		OR	95% CI	*p* value
Model *Δ*3 M-1	*Δ*3M HbA1c (−1%)	1.30	0.92–1.89	0.14

Model *Δ*3 M-2	*Δ*3 M HbA1c (−1%)	0.84	0.43–1.55	0.58
	Baseline HbA1c (+1%)	1.61	0.95–2.97	0.08

Model *Δ*3 M-3	*Δ*3M HbA1c (−1%)	1.15	0.78–1.72	0.48
	Therapy intensification (yes)	2.97	0.73–12.8	0.13

Model *Δ*3 M-4	*Δ*3 M HbA1c (−1%)	0.81	0.40–1.50	0.51
	Baseline HbA1c (+1%)	1.51	0.87–2.83	0.15
	Therapy intensification (yes)	2.38	0.55–10.3	0.25

Model *Δ*6 M-1	*Δ*6 M HbA1c (−1%)	1.24	0.91–1.73	0.17

Model *Δ*6 M-2	*Δ*6 M HbA1c (−1%)	0.81	0.44–1.44	0.46
	Baseline HbA1c (+1%)	1.67	0.94–3.07	0.08

Model *Δ*6 M-3	*Δ*6M HbA1c (−1%)	1.10	0.77–1.58	0.60
	Therapy intensification (yes)	3.19	0.74–14.6	0.12

Model *Δ*6 M-4	*Δ*6 M HbA1c (−1%)	0.75	0.40–1.37	0.36
	Baseline HbA1c (+1%)	1.59	0.88–2.96	0.13
	Therapy intensification (yes)	2.73	0.60–12.4	0.19

Model *Δ*12 M-1	*Δ*12 M HbA1c (−1%)	1.42	1.04–2.03	0.03^*^

Model *Δ*12 M-2	*Δ*12 M HbA1c (−1%)	1.25	0.66–2.50	0.50
	Baseline HbA1c (+1%)	1.16	0.59–2.22	0.66

Model *Δ*12 M-3	*Δ*12 M HbA1c (−1%)	1.29	0.91–1.90	0.17
	Therapy intensification (yes)	2.48	0.57–11.3	0.23

Model *Δ*12 M-4	*Δ*12M HbA1c (−1%)	1.20	0.62–2.43	0.58
	Baseline HbA1c (+1%)	1.09	0.55–2.12	0.81
	Therapy intensification (yes)	2.41	0.54–10.7	0.25

Model baseline HbA1c	Baseline HbA1c (+1%)	1.38	1.02–1.92	0.04^*^

Model therapy intensification	Therapy intensification (yes)	3.21	0.94–11.7	0.06

Model baseline HbA1c + therapy intensification	Baseline HbA1c (+1%)	1.26	0.90–1.80	0.19
	Therapy intensification (yes)	2.59	0.62–11.2	0.19

^*^ *p* < 0.05.

## Data Availability

The data that support the findings of this study are available from Akifumi Kushiyama upon reasonable request.
